# Supplementary figures and images for: Nitrogen Supplementation Modulates Morphological, Biochemical, Yield and Quality Attributes of Peppermint
Source: Plants (Basel). 2023 Feb 10;12(4):809. doi: 10.3390/plants12040809 (PMC9962011; doi:10.3390/plants12040809)

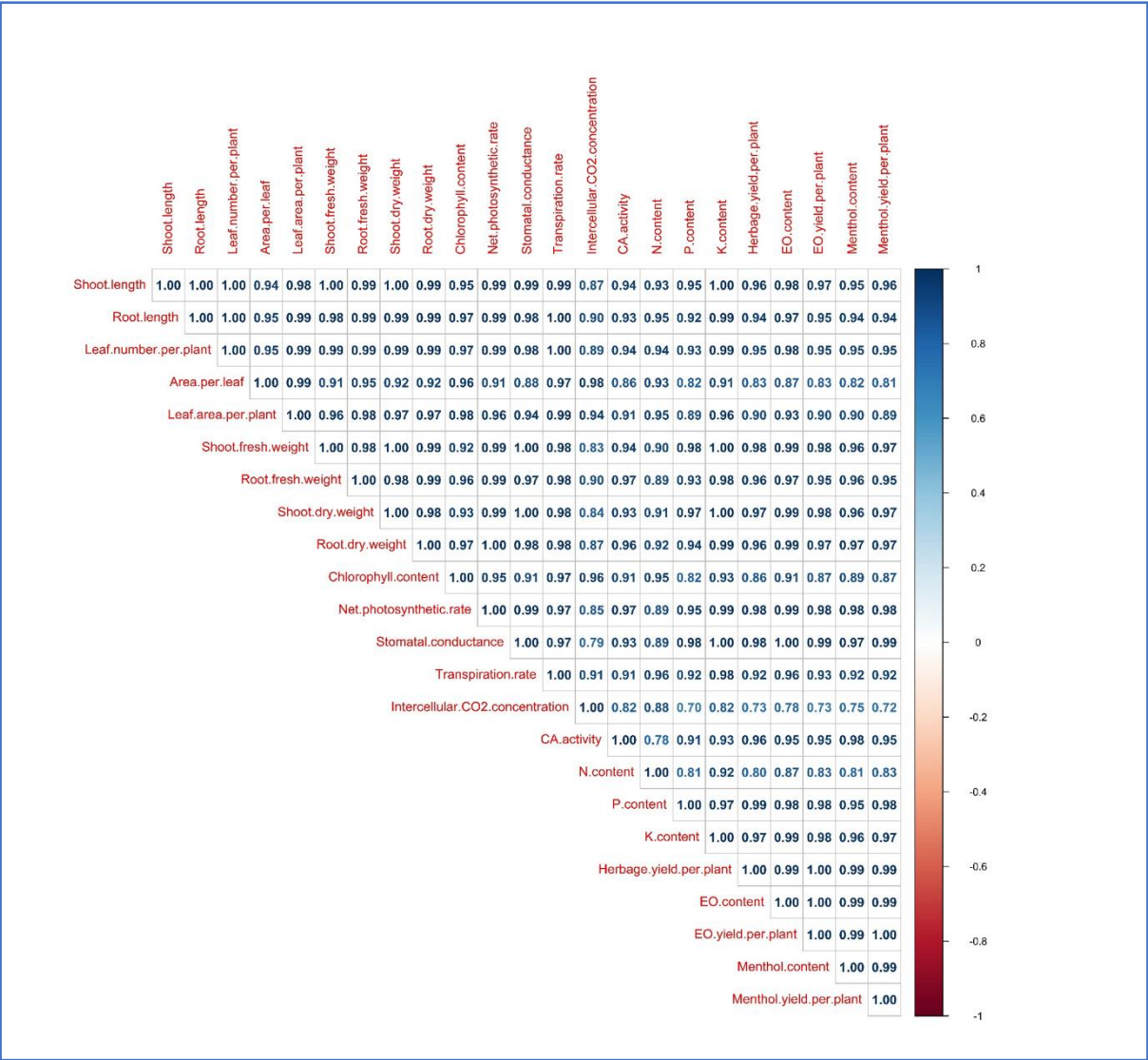

Figure S1.

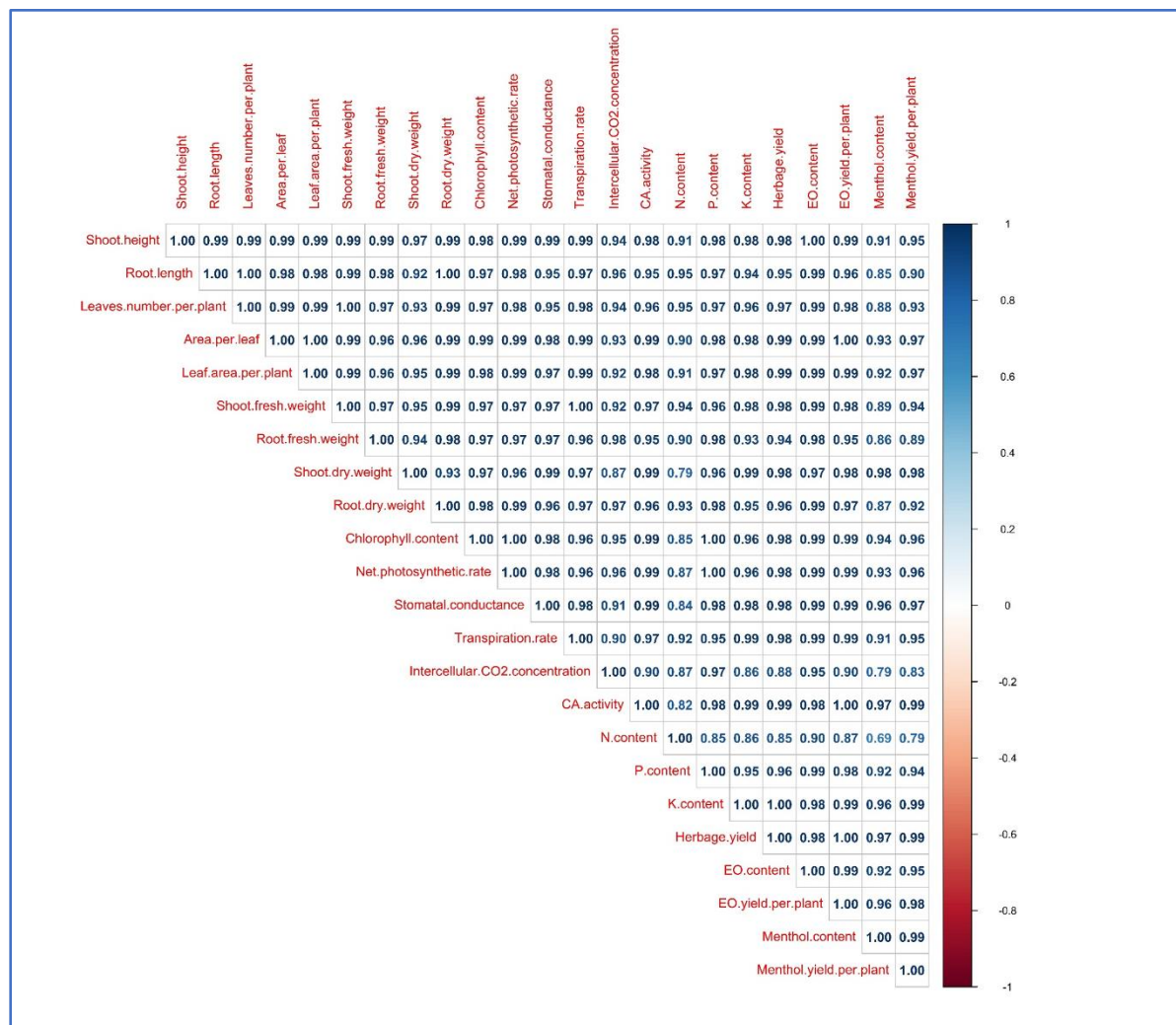

Figure S2.

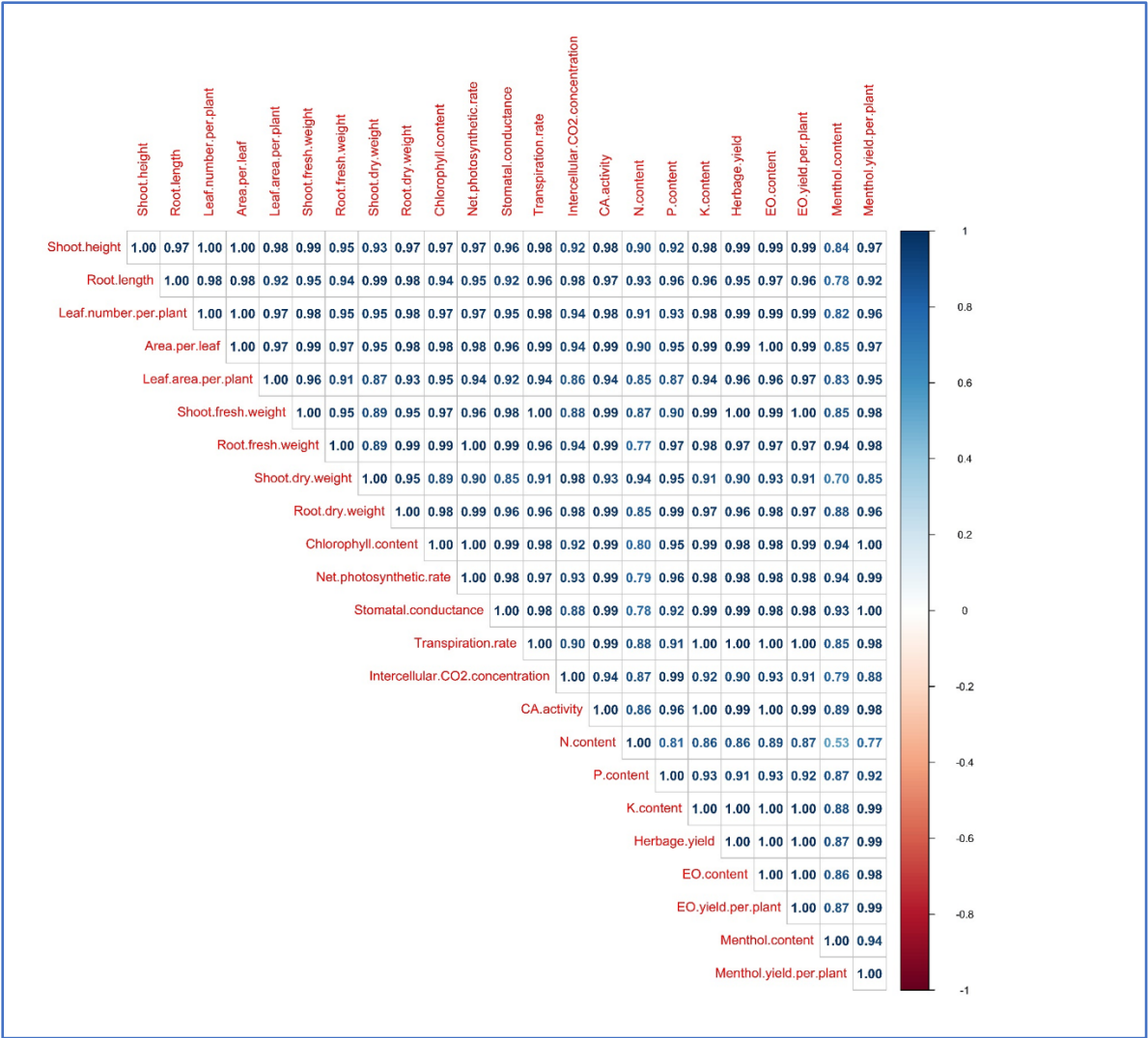

Figure S3.

Supplement: Supplementary file 1 [file plants-12-00809-s001.zip › plants-2201665-supplementary.pdf]
